# The inaugural *mBio* Junior Editorial Board—lessons learned and the path forward toward improving the peer review process

**DOI:** 10.1128/mbio.01991-23

**Published:** 2023-12-15

**Authors:** Cynthia Ayefoumi Adinortey, Stephen K. Dolan, Sarah Doore, Rebeccah Lijek, Diana Priscila Pires, Wenqi Yu, Elizabeth B. Draganova, Lennart Schada von Borzyskowski

**Affiliations:** 1 Department of Molecular Biology and Biotechnology, University of Cape Coast, Cape Coast, Ghana; 2 Department of Genetics and Biochemistry, Clemson University, Clemson, South Carolina, USA; 3 Eukaryotic Pathogens Innovation Center, Clemson University, Clemson, South Carolina, USA; 4 Department of Microbiology and Cell Science, University of Florida, Gainesville, Florida, USA; 5 Department of Biological Sciences, Mount Holyoke College, South Hadley, Massachusetts, USA; 6 Centre of Biological Engineering, Universidade do Minho, Braga, Portugal; 7 Department of Molecular Biosciences, University of South Florida, Tampa, Florida, USA; 8 Department of Biochemistry, Emory University School of Medicine, Atlanta, Georgia, USA; 9 Institute of Biology Leiden, Leiden University, Leiden, the Netherlands; Johns Hopkins Bloomberg School of Public Health, Baltimore, Maryland, USA

## Abstract

The inaugural Junior Editorial Board (JEB) of *mBio* consisted of 64 early-career researchers active from 2022 to 2023. The goal of the JEB was to train early-career researchers in the art of peer review under the guidance of experienced editors. JEB members gained hands-on experience in peer review by participating in modules detailing the publishing process through the lenses of the journal, editor, and reviewer. Ultimately, JEB members applied this new knowledge by reviewing *mBio* manuscripts. Here, we summarize the background, the mission, and the achievements of the first *mBio* JEB. We also include possible trajectories for the future editions of this important program.

## EDITORIAL

### Training peer reviewers is key to improving science publishing

Most researchers consider peer review to be integral to science; it is revered as a critical validation process that differentiates science from pseudoscience (1). Peer review also drives decisions about the research that is published and funded and, in some cases, creates a gatekeeping bottleneck that can make or break careers. Given that peer review is central to the scientific enterprise, it is shocking how little attention is given to training scientists in peer review. A landmark 2019 study of 500 early-career researchers (ECRs), primarily graduate students and postdocs, showed that peer review education is rare: only 11% had formal peer review training, while 25% reported no training (2). Others developed peer review strategies from reading reviews of their own papers or by ghostwriting reviews on behalf of their research advisor, both of which have well-documented flaws (3–8).

Yet not all ECRs have the privilege of receiving peer review training from a Principal Investigator (PI). Furthermore, PIs are typically not trained to provide quality education in peer review. Indeed, the only randomized controlled trial of PI-ECR training dyads showed no benefit over untrained ECRs reviewing alone (9). The issues surrounding the lack of formalized peer review training are compounded by the current reviewer shortage that results in editorial delays (10, 11). Furthermore, the inability to obtain enough reviewers results in manuscript decisions based on only one or two reviews, which can also create more bias in peer review. Therefore, alternative strategies are needed to both increase the number of available peer reviewers and improve the robustness of the peer review process.

A clear solution is to recruit and train more scientists, particularly ECRs, as peer reviewers. ECRs are a diverse, skilled, yet largely untapped source of potential reviewers with a strong desire to gain more expertise in peer review. Several journals have taken advantage of this by establishing training programs designed to include ECRs. These range from free, 1-day online training courses (https://masterclasses.nature.com/focus-on-peer-review-online-course/16605550), to ECR reviewer boards (https://elifesciences.org/inside-elife/eb42df87/early-career-reviewers-pool-authors-can-now-select-and-nominate-early-career-reviewers-for-their-work, https://www.jbc.org/ecr), to mentored, 2-year training programs (https://genetics-gsa.org/career-development/peer-review-training-program/). The latter programs aim to give ECRs real-world peer review experience after defined training sessions.

### Junior editorial boards can offer in-depth ECR training in peer review

**Fig 1:**
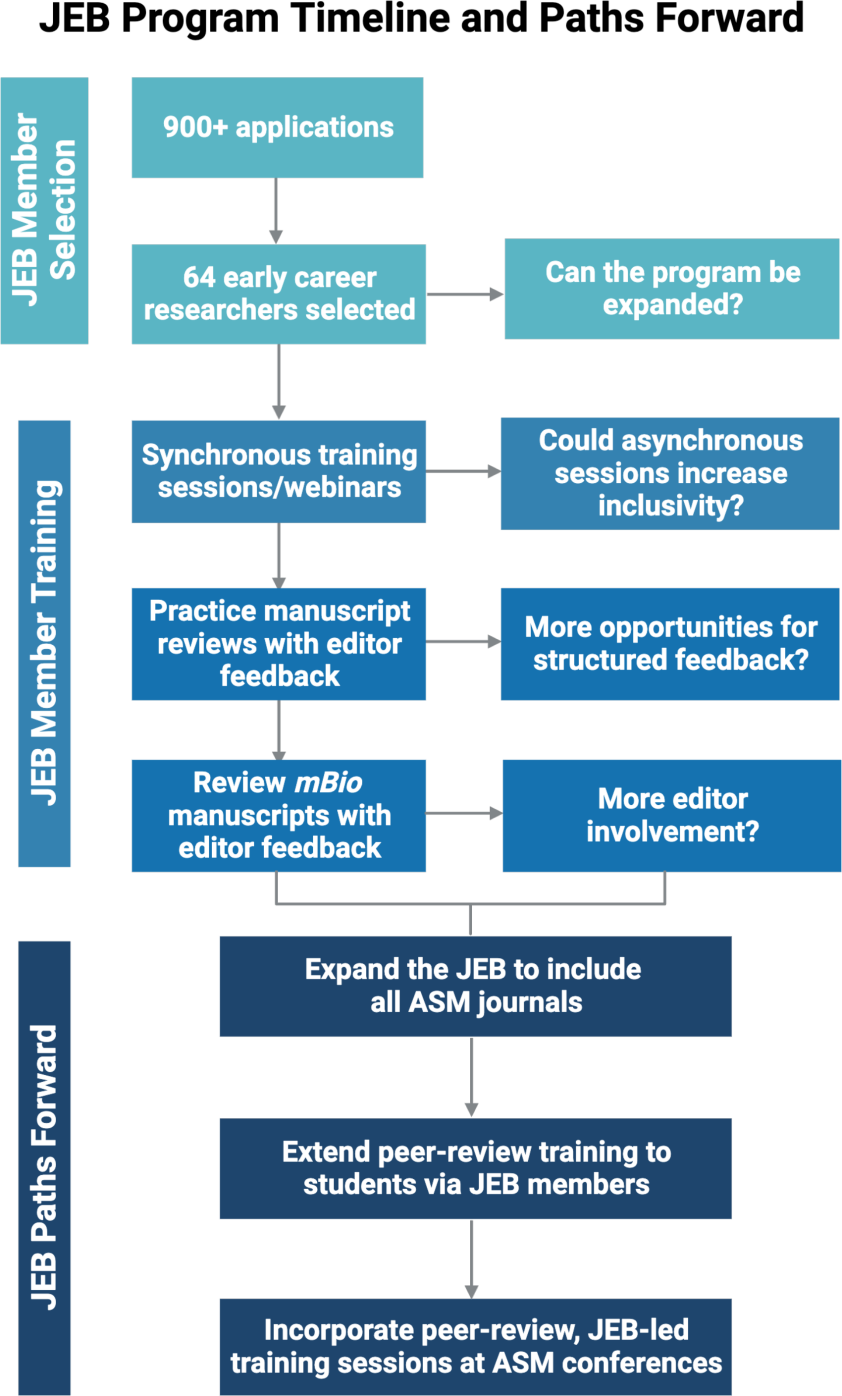
Overview of the JEB reviewer training program and paths forward. The 2022–2023 JEB consisted of 64 members who were trained to review manuscripts for the ASM journal *mBio* through a series of training phases, ultimately leading to independent reviews by JEB members. Suggestions and strategies for improving and expanding the JEB program are listed within the corresponding timeline of the JEB program. This figure was made with BioRender.com.

In January 2022, *mBio* created its inaugural Junior Editorial Board (JEB), consisting of 64 ECRs from around the globe (Fig. 1). The central goal of the JEB was to train ECRs as American Society for Microbiology (ASM) peer reviewers and to provide JEB members with opportunities to review manuscripts submitted to *mBio*. The program has three pillars: training, hands-on experience, and mentor/peer-to-peer collaboration. Initially, monthly training sessions, led by ASM staff, editors, and invited experts, covered various topics, including the publishing process, editor roles, journal selection and predatory journals, a breakdown of the peer-review process, including bias and ethics, and training on ASM activities, including education activities and an introduction to ASM ambassadors.

Hands-on training began with working groups consisting of JEB members and *mBio* editors who, together, practiced “reviewing” published manuscripts. JEB members then transitioned to reviewing manuscripts independently and receiving feedback from editors involved in the review of that manuscript. This type of feedback is essential for improving the quality of ECR manuscript reviews. Between the training sessions and editor feedback, members of the JEB were given opportunities to network with ASM program leaders, editors, and other ECRs. This networking is not only beneficial for advancing ECR career opportunities but also bridges the gap in publishing between the journal staff and the scientists.

Overall, the *mBio* inaugural JEB has highlighted the duality of benefits to these types of training programs between scientists and journals. From a scientist’s perspective, the JEB provided opportunities to not only improve their own peer review process but to also understand how academic publishing operates from the perspectives of the reviewer, editor, and journal staff. In turn, the JEB provided *mBio* with more accessible and trained peer reviewers who were cognizant of the *mBio* publication process. On a larger scale, JEB members will now pass this newfound knowledge on to peers and trainees, having a positive impact on the next generation of scientists. Initiated by this first cohort, the JEB program is an example of a beneficial training program for scientists, the academic peer review system, and the scientific publishing industry.

### The current and future impact of JEBs

With the recruitment of a new cohort of the *mBio* JEB underway, what can be done to improve this important training program? The current *mBio* JEB is centered around a single ASM journal, but would it perhaps be possible to establish a single board that spans across all ASM journals? In this case, ECRs could attend joint training sessions and then be assigned to review manuscripts for a certain journal based on their field-specific expertise. In contrast, a JEB program for each journal means that a group of people are trained specifically on the types of papers that come to that journal. The benefit is that these JEB members become especially familiar with the scope and impact expected for the journal, plus the papers they review are likely of similar caliber and/or may more often be in the same field as their expertise. It will be up to ASM editors and journal staff to make the best choice going forward.

The goals of the JEB could also be expanded to include community service initiatives focused on introducing and training undergraduate and graduate students on the topic of peer review. Advanced undergraduate and new graduate students are often unfamiliar with the process of scholarly publishing and may not understand what exactly goes into the peer review process. Many graduate students participate in journal clubs to hone their peer review skills, but these clubs vary widely in scope and rigor and often depend on the ECR’s own journal club experiences. JEB members could easily translate this knowledge to students and trainees in a structured manner, similar to the current JEB training format, which would result in a graduate-level course on peer review. This could be extended to ASM conferences, where sessions on peer review could be led by JEB members, journal staff, and editors, providing additional opportunities for networking and collaboration.

Overall, the inaugural *mBio* JEB was a successful first step in improving the peer review process. The JEB training program tapped into a network of peer reviewers who, in turn, were trained in the art of peer review. Continued success of this program will involve editor participation, including the selection of ECRs for article review and consistent feedback regarding the quality of manuscript reviews performed by ECRs. The JEB program has certainly created a network of ECR reviewers empowered with the knowledge to educate academic peers and future generations of scientists in peer review.

## References

[1] Baldwin M. 2018. “Scientific autonomy, public accountability, and the rise of “peer review” in the cold war United States”. Isis 109:538–558. doi:10.1086/700070

[2] McDowell GS, Knutsen JD, Graham JM, Oelker SK, Lijek RS. 2019. Co-reviewing and ghostwriting by early-career researchers in the peer review of manuscripts. eLife 8. doi:10.7554/eLife.48425 PMC6822987 31668163

[3] Dance A. 2023. Stop the peer-review treadmill. I want to get off. Nature 614:581–583. doi:10.1038/d41586-023-00403-8 36781962

[4] Dance A. 2022. Why early-career researchers should step up to the peer-review plate. Nature 602:169–171. doi:10.1038/d41586-022-00216-1 35102332

[5] Flaherty C. 2019. Ghostwriting peer reviews. Inside Higher Ed. Available from: https://www.insidehighered.com/news/2019/11/01/ghostwriting-peer-reviews-advisers-more-common-you-might-think

[6] Akst J. 2019. Trainees often ghostwrite PIs’ peer reviews: survey. The Scientist. https://www.the-scientist.com/news-opinion/trainees-often-ghostwrite-pis-peer-reviews--survey-66675.

[7] Benderly BL. 2019. Early-career researchers commonly ghostwrite peer reviews. That’s a problem. Science. doi:10.1126/science.caredit.aax9372

[8] Gewin V. 2019. Junior researchers are losing out by ghostwriting peer reviews. Nature. doi:10.1038/d41586-019-01533-8 32393872

[9] Houry D, Green S, Callaham M. 2012. Does mentoring new peer reviewers improve review quality? a randomized trial. BMC Med Educ 12:83. doi:10.1186/1472-6920-12-83 22928960 PMC3494517

[10] Petrescu M, Krishen AS. 2022. The evolving crisis of the peer-review process. J Market Anal 10:185–186. doi:10.1057/s41270-022-00176-5

[11] Flaherty C. 2022. The peer-review crisis. Inside Higher Ed. Available from: https://www.insidehighered.com/news/2022/06/13/peer-review-crisis-creates-problems-journals-and-scholars

